# Agility in humanitarian supply chain: an organizational information processing perspective and relational view

**DOI:** 10.1007/s10479-020-03824-0

**Published:** 2020-10-23

**Authors:** Rameshwar Dubey, David J. Bryde, Cyril Foropon, Gary Graham, Mihalis Giannakis, Deepa Bhatt Mishra

**Affiliations:** 1 Liverpool Business School, Liverpool John Moore’s University, Liverpool, Merseyside, L3 5UG UK; 2 Montpellier Business School, Montpellier Research in Management, 2300 Avenue des Moulins, 34185 Montpellier, France; 3 Leeds University Business School, University of Leeds, Maurice Keyworth Building, Leeds, LS2 9JT UK; 4 Audencia Business School, Nantes, France

**Keywords:** Collaboration, Humanitarian supply chain, Information sharing, Organizational information processing theory, Relational view, Supply chain visibility, Swift-trust

## Abstract

Humanitarian organizations are increasingly facing challenges in terms of improving the efficiency and the effectiveness of their disaster relief efforts. These challenges often arise due to a lack of trust, poor collaboration and an inability to respond to disaster affected areas in a timely manner. Our study attempts to understand how these challenges are overcome by seeking answers to questions related to the topics of swift-trust, collaboration and agility in humanitarian supply chains. For instance, in our study we have attempted to examine how information sharing and supply chain visibility in humanitarian supply chains improve the swift-trust among the humanitarian actors engaged in disaster relief operations. Further, we attempt to understand how-swift trust, commitment and collaboration among the humanitarian actors improve the agility in humanitarian supply chains. In our study we provide both theoretical and data-driven answers to our stated research gaps. Our theoretical model is firmly grounded in organizational information process theory and relational view. We tested our research hypotheses using variance based structural equation modelling with survey data collected using a web based pre-tested instrument from 147 NGOs respondents drawn from the National Disaster Management Authority database. Our results help to advance the theoretical debates surrounding “swift-trust”, “collaboration” and “agility” in humanitarian settings. We further provide direction to managers engaged in disaster relief operations. The humanitarian actors engaged in disaster relief often fail to understand how to build swift-trust. Moreover, how swift-trust further affects commitment and collaboration which in turn further affect agility in humanitarian supply chains. Thus humanitarian organizations must understand how information sharing and supply chain visibility is key to swift-trust among humanitarian actors and agility in humanitarian supply chains. Finally, we outline the limitations of our study and offer some future research directions for investigation.

## Introduction

Due to the rapid rise in disasters resulting from climate change there are serious challenges being posed for humanitarian organizations engaged in managing disaster relief supply chains (Papadopoulos et al. 2017; Dubey et al. 2019c; Jabbour et al. 2019; Fosso Wamba 2020). It may be argued that the guiding principles of humanitarian logistics (i.e. right goods or service, right place, right time, and right condition) are critical factors of success. However unlike commercial supply chains, disaster relief teams typically do not have all the necessary information to ensure the humanitarian supply chain works effectively and efficiently, such as knowledge of the needs of survivors or useful alternative routes to administer aid in disaster-hit locations (Seybolt 2009; Swanson and Smith 2013; Behl and Dutta 2019; Gupta et al. 2019; De Camargo et al. 2019; Ivanov and Dolgui 2020).

Due to the unpredictable nature of the events relating to a disaster, humanitarian supply chains are often hastily formed (Tatham and Kovács 2010; Dubey et al. 2019a; Queiroz et al. 2020; Wagner et al. 2020; Modgil et al. 2020). Hence, designing a humanitarian supply chain is a far more complex activity relative to designing a commercial supply chain (Holguín-Veras et al. 2012; Banomyong et al. 2019; Shareef et al. 2019; Stewart and Ivanov 2019). For example, whenever a disaster hits a region, a considerable number of relief organizations, host governments, the military, local and regional relief organizations, and private sector organizations, each with different mandates, interests, capacity and capabilities, are involved (Balcik et al. 2010). Such complexity is usually not present in commercial supply chains.

Oloruntoba and Kovács (2015) argue that humanitarian-related activities must increase their responsiveness and flexibility to meet dynamic humanitarian needs due to disasters. Yet typically no single organization has sufficient resources and capabilities to respond effectively to a major disaster (Altay et al. 2018). A topic that has gained increased attention as a possible means of enhancing responsiveness and flexibility in organizational contexts is that of agility and its beneficial role in humanitarian supply chain has gained increasing popularity among the scholars engaged in non-profit supply chains design (see, Oloruntoba and Gray 2006). Indeed Dubey and Gunasekaran (2016) argue that agility in humanitarian supply chains is a desired capability that helps organizations to thrive and prosper in dynamic and uncertain environments. Oloruntoba and Kovács (2015) further argue that the agile humanitarian supply chain is vital for enhancing the effectiveness of disaster relief aid. Whilst there is a rich body on humanitarian supply chain agility (Oloruntoba and Kovács 2015; Dubey and Gunasekaran 2016; L’Hermitte et al. 2016; Kabra and Ramesh 2016; Altay et al. 2018; Ivanov 2020), research on *when* and *how* humanitarian organizations create agility in humanitarian supply chain is very limited. Existing studies on humanitarian supply chain agility mainly focuses on ascertaining the organizational impact of humanitarian supply chain agility in terms of humanitarian operations performance. Few studies utilize a theory-driven approach to understand the role of key factors in building humanitarian supply chain agility (Gunasekaran et al. 2018). We identify this as a clear research gap in humanitarian supply chain literature. Hence, based on preceding discussions we posit our guiding research question as:

- RQ1: What are the antecedents of agility in humanitarian supply chains?

Chen et al. (2011, p. 262) state that “commitment involves continuity or a long-term orientation with both parties cooperating to maintain the relationship”. Further, they argue that commitment amongst the participating organizations helps increase efficiency and effectiveness. Christopher (2000) further argues that commitment and collaboration are essential building blocks of the agile supply chain. Following on from these arguments and from Morgan and Hunt’s (1994) tenets, we posit that trust and commitment stimulate a relational bond between humanitarian actors that facilitates the collaboration amongst the humanitarian actors involved in relief operations (Dubey et al. 2019a). Drawing upon on organizational information processing theory perspective (OIPT) (Smith et al. 1991; Gattiker and Goodhue 2004; Srinivasan and Swink 2015, 2018; Dubey et al. 2019b, 2020) and relational view (RV) literature (Dyer and Singh 1998; Wieland and Wallenburg 2013; Chen et al. 2013; Moshtari 2016) we develop a model to help understand how information sharing and supply chain visibility, an important activity and characteristic, respectively, of effective supply chains, helps to quickly build trust among disaster relief supply chain actors, which further leads to enhanced commitment and collaboration and, finally, to agility (Dubey et al. 2019a). Whilst it is reasonable to assume that commitment and collaboration are critical elements for building agility in humanitarian supply chains, we posit that theoretical explanations regarding the effects of these factors on agility are still largely underdeveloped. There is a need for theory-driven empirical study which focuses on commitment and collaboration and their effects on agility, which our paper meets. Hence, we identify this as a clear research gap. We posit our guiding research question as:

- RQ2: How these antecedents affects the agility in humanitarian supply chains?

By developing and empirically testing our theoretical model, our study offers two main contributions to knowledge on humanitarian operations management. Firstly, we demonstrate the extent to which information sharing and supply chain visibility influence the swift-trust among the actors engaged in disaster relief operations. Secondly, we enhance understanding of how organizations build agility in humanitarian supply chains via enhancing commitment and collaboration among disaster relief actors. Our findings provide theory development in relation to OIPT and RV, by testing each in the context of different actors in the supply chains providing humanitarian aid to disaster relief.

The remainder of our paper is organized as follows. The next section presents the guiding theoretical framework. Here, drawing from the OIPT and RV literature we develop our theoretical framework, which shows hypothesized relationships between *information sharing, supply chain visibility, swift-trust, commitment, collaboration* and *agility*. In the third section we outline the research design. We derive from the extant literature the constructs used in the framework and describe our survey-based method used to collect data from 147 actors involved in disaster relief activities on the Indian sub-continent to measure the constructs. We report on the pre-testing and data collection procedure and the results of non-response biasness testing, which shows that such bias is not evident. The fourth section deals with our data analyses, in which we utilize variance-based structural equation modelling (SEM), with Partial Least Squares (PLS), to test the hypotheses. We report the assumptions test, confirmatory factor analysis and a goodness of fit test, which confirm the robustness of the model. The fifth section discusses our statistical analyses results, theoretical contributions and managerial implications. Here we highlight how integration of OIPT and RV viewpoints provides a useful lens to understand how agility is built up in humanitarian supply chains. Such understanding enables actors to swiftly build trust in the supply chain, which is an important precursor of agility. Finally, we present our conclusions from the research investigation, in which we emphasize how our theoretical framework can guide steps to improve agility. We also outline the key limitations of our study, in respect of generalizing the findings beyond the empirical case of India and hence, we end by recommending further data collection from other international contexts, along with alternative methodological approaches to collect data and test the framework.

## Theoretical framework and research hypotheses

**Fig. 1:**
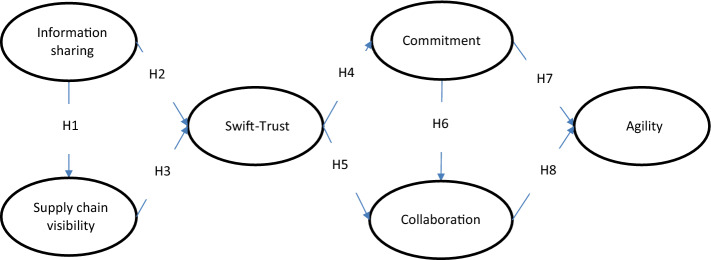
Theoretical framework

We have looked at a wide range of literature from across different management disciplines to extract the constructs in the theoretical framework shown in Fig. 1. Our framework is grounded in OIPT and RV. Organizations must use the information they collect effectively, especially when they are facing a degree of uncertainties (Galbraith 1973; Srinivasan and Swink 2018). Following Galbraith’s (1973) arguments organizations generally have two choices in respect of their information-related approaches: firstly, organizations may reduce their needs for information via mechanistic organizational structures. Secondly, organizations may try to enhance their information processing capabilities. Regardless of approach, there is a need to share information, both vertically up and down hierarchies and horizontally between different business units and functions. Hence, we argue that information sharing is a vital activity undertaken by any organization to gain competitive advantage and is crucial to the efficient and effective operation of supply chains.

We consider supply chain visibility as an important organization capability in reducing risk (Brandon-Jones et al. 2014) and enhancing swift*-*trust (Dubey et al. 2018), yet, surprisingly, empirical evidence of its effects is largely missing from the literature. Visibility is an important antecedent to cooperation and agility in the supply chain (Lee et al. 1997; Wang and Wei 2007; Brusset 2016).

In our study we seek to investigate how information sharing among partners in humanitarian supply chain enhances visibility. The RV suggests that organizations can obtain competitive advantages via relational rents or benefits that are generated via collaborative relationships (Dyer and Singh 1998; Cao and Zhang 2011). Whilst OIPT posits that a key task of the organisation is resolving uncertainty, where the source of uncertainty is a lack of information (Gattiker and Goodhue 2004; Galbraith 1973). Relying on previous research (Morgan and Hunt 1994; Moshtari 2016; Dwivedi et al. 2018; Dubey et al. 2019a), we advance the proposition that relational orientations (i.e. swift-trust and commitment) leads to improved collaboration among partners engaged in humanitarian supply chains. Hence, following tenets of OIPT and RV we propose a theoretical framework (see Fig. 1).

In the next sections we elaborate on the constructs used in our framework and the hypothesized relationships between them.

### Information sharing and supply chain visibility

Information is an intangible resource and should be timely, full, correct, pertinent and confidential (Cao and Zhang 2011). The importance of information sharing in supply chains has gained significant recent attention, due to the rapid growth in digitization (Fawcett et al. 2007; Prajogo and Olhager 2012; Brandon-Jones et al. 2014; Gunasekaran et al. 2017). Information sharing can be defined as an organizational capital, a resource which focuses on the flow of information (Premkumar and King 1994; Altay and Pal 2014) have argued the need for information diffusion [sharing] among agents to improve response in disaster relief supply chain networks. Brandon-Jones et al. (2014) further argue that sharing the right information at the right time among supply chain actors may lead to improved visibility in supply chain networks, especially if related to information sharing in the context of inventory and demand levels across the supply chain Tang 2006; Barratt and Oke 2007; Sodhi and Tang 2019; Dubey et al. 2019a). Hence, we hypothesize the relationship between information sharing and supply chain visibility as follows:

#### **H1**

Information sharing amongst humanitarian actors has a positive impact on supply chain visibility.

### Information sharing, supply chain visibility and swift-trust

During disaster relief operations, the main goals of humanitarian organizations is to carry out humanitarian relief efforts and assume responsibilities for minimizing the negative effects of disaster and economic reconstruction (Dubey et al. 2019b; Schiffling et al. 2020a, b). Due to a high degree of uncertainty, the humanitarian actors temporarily engage in relief efforts and other aspects of the reconstruction phase to provide support to the victims and restore normality (Jabbour et al. 2019; Cankaya et al. 2019). These situations, which are complex environments in which to operate (Dubey et al. 2019b) require the careful managing of information among humanitarian actors (Altay and Pal 2014; Altay and Labonte 2014; Dubey et al. 2019a; Akter and Wamba, 2019). Achieving a shared humanitarian organizations’ vision, managing shared expectations, sharing information, improving visibility, and improving collaboration are crucial factors for efficient and effective disaster relief operations (Altay and Labonte 2014; Dubey et al. 2019b).

It is often understood that humanitarian organizations with high visibility and effective information-sharing capabilities are well positioned to collaborate in highly uncertain and complex environments Prasad et al. 2018; Oloruntoba et al. 2019; Fosso Wamba et al. 2019). We can further argue that those humanitarian organizations that invest in developing information sharing capabilities are also most likely to invest in supply chain visibility capability, because visibility further improves swift-trust (Dubey et al. 2018, 2019b). A lack of swift-trust is often considered as a major source of conflict amongst the humanitarian actors engaged in disaster relief efforts (Tatham and Kovács 2010; McLaren and Loosemore 2019; Dubey et al. 2019a).

Dubey et al. (2019b) state that for effective collaboration it is important to understand the roles, relationships, capabilities, motivations, and information-sharing needs in complex environments. Thus, we can argue that information sharing and supply chain visibility are complementary, in the sense that they each demand and support each other. Swift-trust emerges from information sharing and supply chain visibility (Dubey et al. 2018). Hence, humanitarian organizations need to understand the connections between analytics capability, swift trust, and collaborative performance. Following this line of reasoning, we posit the hypotheses:

#### **H2**

Information sharing amongst humanitarian actors has a positive impact on swift-trust.

#### **H3**

Supply chain visibility has a positive impact on swift-trust.

### Swift-trust, commitment and collaboration

Conway and Swift (2000) have identified trust and commitment as the two most important factors for building relationships among supply chain partners, with trust a precondition for building commitment (Morgan and Hunt 1994; Wilson 1995) further supports the argument of Morgan and Hunt (1994) by identifying trust as an important building block in the development of a relationship framework; with commitment of central importance in relationship management. Kwon and Suh (2004) further argue that trust is an integral component of commitment, which is a desirable property for improving coordination in a supply chain network. Building on the work of Hocutt (1998) we define commitment as an intention to continue a course of action or activity.

Given that humanitarian supply chains are often hastily formed (Tatham and Kovács 2010), the concept of “swift*-*trust” is highly relevant. Meyerson et al. (1996) coined the term “swift-trust” which is essential for bringing together temporary teams formed with a clear purpose and common task with a finite life span. Hence, we argue that swift-trust may have a positive impact on the commitment of actors in the humanitarian supply chain network. Humanitarian operations face challenges resulting from the diversity of humanitarian actors engaged in disaster relief operations (Moshtari 2016). As a response to this challenge, Prasanna and Haavisto (2018) argue that openness and honesty helps build trust and commitment amongst these diverse humanitarian actors engaged in disaster relief operations. Furthermore, it is suggested that trust and commitment can further enhance collaboration between different humanitarian actors (Prasanna and Haavisto 2018; Wagner and Thakur-Weigold 2018; Dubey et al. 2019b). Hence, we hypothesize the following:

#### **H4**

Swift trust has a positive impact on commitment of humanitarian supply chain actors.

#### **H5**

Swift-trust has a positive impact on collaboration.

#### **H6**

Commitment has a positive impact on collaboration.

### Commitment, collaboration and agility in humanitarian supply chain network

We define organizational agility based on critical review of literature (see, Sambamurthy et al. 2003; Blome et al. 2013; Dubey and Gunasekaran 2016) as “an organization’s ability to detect changes, opportunities, and threats in its business environment and to provide speedy and focused responses to customers and other stakeholders by reconfiguring resources and processes and/or by developing strategic partnerships and alliances” (Sambamurthy et al. 2003). Additionally, some scholars perceive organizational agility as an extension of organizational flexibility, allowing the organization to embed enabling mechanisms into its intra- and inter-organizational processes and IT systems. Thereby, responding to both the predictable changes in the marketplace (Lu and Ramamurthy 2011) and providing the organizational strategic flexibility that is necessary when handling unstructured changes (Hitt et al. 1998; Lu and Ramamurthy 2011). In disaster affected areas, which are characterized by a chaotic, hypercompetitive environment and a high level of uncertainty, supply chain agility is considered as an important determinant of organization success (Dubey and Gunasekaran 2016).

Agility in humanitarian supply chain networks has attracted significant attention from operations management scholars (see, Charles et al. 2010; Cozzolino et al. 2012; Day et al. 2012; Altay et al. 2018). Charles et al. (2010) argue that flexibility is the foundation of agility. Characterized by its ability to sense any changes in the internal and external environment, it involves flexibility to respond to any changes and speed with which an organization can respond to any changes in the environment (Blome et al. 2013). Dubey et al. (2019a, b, c) further argues that trust and commitment are important elements of a participative management style and important enablers of an agile supply chain (Sherehiy et al. 2007; Lee 2004) adds to the discourse by suggesting that collaboration is an important element in an agile supply chain network, where that collaboration can be achieved through building commitment (Yusuf et al. 2014). Hence, we posit that commitment is an essential element of collaboration; and further, collaboration, built through commitment, is an important element of agility (see, Narayanan et al. 2015). Therefore we hypothesize these relationships as follows:

#### **H7**

Commitment has a positive impact on agility.

#### **H8**

Collaboration has a positive impact on agility.

## Research method and data

### Construct operationalization

To test our theoretical framework, shown in Fig. 1, we have used the survey method. A measuring instrument was developed by identifying appropriate constructs and items via a critical review of existing literature. We have made modifications to the existing scales to make our measurements appropriate to the context of humanitarian supply chains, since most of the measurement scales were developed in the context of commercial supply chains. A panel of experts involved in disaster relief activities from state government, military, and NGO’s examined the face validity of the items. In the next sections we explain the modifications made to adapt the existing scales to the context of humanitarian supply chain networks.

#### Information sharing

As explained above, information sharing has been identified as one of the important factors in successful supply chain management (Yu et al. 2001; Kwon and Suh 2004; Li and Lin 2006; Sezen 2008; Vijayasarathy 2010). We reviewed pertinent literature as a basis for developing this scale i.e. Zhou and Benton (2007); Hsu et al. (2008); Yigitbasioglu (2010). We particularly refer to Balcik et al. (2010) in which they argue that co-ordination among humanitarian supply chain actors refers to resource and information sharing, centralized decision-making, conducting projects, regional division of tasks, or a cluster-based system in which each cluster represents a different sector area (e.g., food, water and sanitation, and information technology). have modified the Hsu et al. (2008) construct to the context of humanitarian supply chains. Hence we have derived three items to measure information sharing: (1) the use of compatible information systems with various actors engaged in disaster relief activities; (2) the sharing of information related to various resources deployed for relief activities i.e. relief materials, manpower, modes of transportation etc.; (3) the existence of a joint information center (JIC) for effective sharing of information among various agencies or organizations involved in a disaster relief project.

#### Supply chain visibility

Following Barratt and Barratt (2011), we conceive how visibility in the supply chain is created through external relations. We further understand how using information systems, effective planning processes and coordinated decision making helps improve visibility in the supply chain (Srinivasan and Swink 2018). Disaster relief workers often seek to improve the visibility of both demand and supply information (Dubey et al. 2018). We further reviewed several works on measures of visibility, see Wang and Wei (2007), Caridi et al. (2010), Maghsoudi and Pazirandeh (2016) and Srinivasan and Swink (2018). Informed by this prior literature we developed three items to measure supply chain visibility: the level of shared understanding of and access to the product related information that they request without loss, noise and distortion (Maghsoudi and Pazirandeh 2016); the extent to which they have on-hand information related to demand and supply for management planning and control (Dubey et al. 2018); and the extent to which they have information related to inventory of relief items and can track the movement of relief items in disaster relief chain (Maghsoudi and Pazirandeh 2016; Dubey et al. 2018).

#### Swift trust

Tatham and Kovács (2010) argue that “swift-trust” has a positive impact on building coordination among humanitarian supply chain actors. To measure swift trust, have reviewed extant literature i.e. Hung et al.( 2004); Tatham and Kovács (2010); Dubey et al. (2019a), we used five items: (1) information regarding actors involved in disaster relief activities; (2) dispositional trust; (3) the clear rule for classification of processes and procedures; (4) role clarity; and (5) classification category i.e. gender, ethnicity etc.

#### Commitment

Morgan and Hunt (1994, p. 23) define commitment as “… an exchange partner believing that an ongoing relationship with another is so important as to warrant maximum efforts at maintaining it; that is, the committed party believes the relationship endures indefinitely”. Kwon and Suh (2004, 2005) state that commitment is central to all relationship management practices and subsequent authors argue that it is vital for supply chain integration (SCI) (Jin et al. 2013). In order to measure commitment. Wu et al. (2004) provide useful findings in relation to the factors that influence commitment in a supply chain network. We have modified Morgan and Hunt’s (1994) construct for measuring commitment, developed in the field of relationship marketing. We therefore have three items to measure commitment: (1) the impact of relationship termination on the ultimate goal of humanitarian supply chain network; (2) the improvement in coordination; (3) shared values.

#### Collaboration

Collaboration may occur on one or more tasks within the disaster relief chain via information exchange, capacity analysis, needs evaluation, resource mobilization, procurement, transportation, storage and the last-mile delivery of relief items to the affected victims (Moshtari 2016). We have reviewed several literatures on measures of collaboration (see, Cao and Zhang 2011; Simatupang and Sridharan 2002, 2011), but they mainly focus on the collaborative relationship among actors in commercial supply chain. We have also adapted several items from Moshtari (2016) and Herlin and Pazirandeh (2012, 2015). Hence, we derived three items as follows: (1) the objectives for which the collaboration established are being met; (2) the engaged humanitarian actors seems to be happy with the overall efforts of the actors towards disaster relief efforts; (3) our humanitarian organization seems to be satisfied with the overall outcome of our collaborative efforts.

#### Agility

Oloruntoba and Gray (2006) argue for the need to build agility in humanitarian supply chain networks, in order to move relief materials efficiently and effectively to the disaster affected locations. The humanitarian supply chain is particularly short lived and quite unstable. Hence in the absence of long-term planning, humanitarian supply chains must possess speed and flexibility if they are to successfully respond to the disaster affected victims with the necessary humanitarian aid, which includes health, food, water and sanitation, shelter and non-food items and other infrastructure needs. Charles et al. (2010) have attempted to build supply chain agility theory to explain a humanitarian approach. Charles et al. (2010) identified five dimensions for agile supply chain: flexibility, velocity, reactivity, visibility and effectiveness. Dubey and Gunasekaran (2016) developed three measures of agility in humanitarian supply chain networks: (1) dynamic sensing, (2) dynamic speed and (3) dynamic flexibility, which are highly appropriate to our study and hence we have used these three items to operationalize the agility construct.

### Data collection

We analyzed data gathered in 2017, via a survey, following protocols established in a previous study, see, Dubey et al. (2019a). We collected data through an institutional collaboration of the NDMA (National Disaster Management Authority). The NDMA is “an agency under the Ministry of Home Affairs (MHA) that was created through the Disaster Management Act in the year 2005, to coordinate responses to natural or man-made disasters and for capacity building to develop resilience in disaster relief supply chain networks and also to improve response to disaster-affected locations” (Dubey et al. 2019a, p. 165). We drew our sample from the NDMA directory of NGOs involved in humanitarian activities. The NDMA Executive Secretary assisted in distributing our questionnaire amongst selected organizations. Of 572 questionnaires distributed, 167 questionnaires were returned, of which 147 were complete and deemed usable for data analysis (see, Akter et al. 2011; Cohen 1992). This gives an effective response rate of 25.69%. We present the demographic profile of the respondents in Table 1.

**Table 1:** Demographic profiles of the respondents

Organization and gender split of respondents	Level of respondent	Number of respondents	Percent of our sample
NGOs	Vice President	26	17.69
	General Manager	37	25.17
	Senior Manager	47	31.97
	Manager	15	10.20
	Deputy Manager	12	8.16
	Assistant Manager	10	6.80

All respondents held managerial positions in their organizations (Vice President, 17.69% of the sample; General Manager, 25.17%; Senior Manager, 31.97%; Manager, 10.20%; Deputy Manager, 8.16% & Assistant Manager, 6.80%).

### Non-response biasness test

Chen and Paulraj (2004) argue that the non-response bias test is one of the main requirements for validating data from statistical surveys, as there is a possibility that the response of the early respondents may differ from that of later respondents (Armstrong and Overton 1977). Hence before data is used for further statistical analyses, it is advisable to conduct a non-response bias test using wave analysis. In this approach, depending upon the nature of the data distribution, either the chi-square test or the t-test is performed on early responses and late responses to check whether a significant statistical difference exists. In recent years there is increasing trends among the operations management research community to use wave analysis to check for non-response bias (see, Blome et al. 2013; Yang 2014; Dubey and Gunasekaran 2016). Following this trend, we split our collected data into two equal halves, as suggested by Chen and Paulraj (2004), depending on the date they were received. Assessing non-response bias test on two halves, using t-tests, we found no significant differences (*p* = 0.26) between the two groups. Hence, we concluded that non-response bias is not a serious issue in our study.

## Data analyses and results

We used Warp PLS 7.0, which relies on the variance-based structural equation modelling (Partial Least Squares) method, as the tool to examine the hypothesized relationships in our theoretical framework (Kock 2019). Following Peng and Lai (2012) we utilize PLS as a prediction-oriented approach that allows us to assess the predictive validity of the exogenous variables. Hence, using PLS enables meeting our study aims, which are (1) to examine the predictive behavior of information sharing and supply chain visibility to further our understanding regarding the building up of swift-trust (see, Dubey et al. 2018, 2019a) and (2) to explain the prediction behavior of commitment and collaboration on building agility in humanitarian supply chain (Narayanan et al. 2015). Although previous studies have tested these hypothesized relationships our purpose is to understand how swift-trust, commitment and collaboration predict agility in the humanitarian supply chain. OIPT and RV are not utilized in organizational literature to examine these relationships, therefore, having no previous theoretical foundation for informing such relationships (see Fig. 1), PLS is a particularly appropriate method for statistical analyses (see, Akter et al. 2011; Peng and Lai 2012; Moshtari 2016; Akter et al. 2017; Dubey et al. 2019b; Motamarri et al. 2020). Finally, we have followed suggestions offered by Peng and Lai (2012) in order to estimate our theoretical model, involving a two stage process: firstly, examining the validity and the reliability of the theoretical model and, secondly, analyzing the structural model.

### Measurement model

We examined the measurement model by assessing constructs’ individual-item reliabilities, the convergent validity of the measures associated with each construct and their divergent validity. We note that all the reliability coefficients are above 0.70, the standardized factor loading of each item is above 0.5, the composite reliability is above 0.5 and each average variance extracted (AVE) is above 0.5 (see Table 2). This indicates that the measurements are consistent, with the latent construct accounting for at least 50% of the variance in the items. Hence, it is evident that our measurement model (see Fig. 1) demonstrates convergent validity. Table 3 shows that the square root of the AVE in the leading diagonal is greater than all the entries in the given row and column (i.e. above correlation coefficient values). These results further suggest that our model possesses divergent validity.

**Table 2:** Loadings of the indicator variables (composite reliability) and average variance extracted (AVE) (N = 147)

Construct	Indicator	Factor loading	Variance	Error	SCR	AVE
IS	IS1 Compatible information systems	0.76	0.58	0.42	0.86	0.67
	IS2 Information sharing	0.84	0.71	0.29		
	IS3 Joint information center	0.85	0.72	0.28		
SCV	SCV1 Product related information	0.66	0.44	0.56	0.83	0.63
	SCV2 information related to demand and supply	0.85	0.72	0.28		
	SCV3 Information related inventory	0.85	0.72	0.28		
ST	ST2 dispositional trust	0.79	0.62	0.38	0.90	0.70
	ST3 Clear rule for classification of process and procedures	0.91	0.83	0.17		
	ST4 role clarity	0.84	0.71	0.29		
	ST5 category	0.79	0.63	0.37		
C	C1 Impact of relationship termination	0.78	0.61	0.39	0.84	0.64
	C2 Improvement in coordination	0.93	0.86	0.14		
	C3 Shared values	0.66	0.44	0.56		
CO	CO1 The objectives for which collaboration was established are being met	0.81	0.66	0.34	0.85	0.65
	CO2 the engaged humanitarian actors seems to be happy with the overall efforts of the actors towards disaster relief efforts	0.75	0.56	0.44		
	CO3 our humanitarian organization seems to be satisfied with the overall outcome of our collaborative efforts	0.76	0.72	0.28		
AG	AG1 Dynamic sensing	0.94	0.88	0.12	0.87	0.71
	AG2 Dynamic speed	0.93	0.86	0.14		
	AG3 Dynamic flexibility	0.61	0.37	0.63		

*IS* information sharing, *SCV* supply chain visibility, *ST* swift-trust, *C* commitment, *CO* collaboration, *AG* agility

**Table 3:** Correlations among major constructs (N = 147)

	IS	SCV	ST	C	CO	AG
IS	**0.82**					
SCV	0.31	**0.74**				
ST	0.08	− 0.16	**0.84**			
C	− 0.09	-0.18	0.13	**0.80**		
CO	0.26	0.17	0.03	0.13	**0.81**	
AG	− 0.02	− 0.02	− 0.12	0.15	0.06	**0.84**

*IS* information sharing, *SCV* supply chain visibility, *ST* swift-trust, *C* commitment, *CO* collaboration, *AG* sgility

The square root of AVE is shown in bold on the diagonal

### Common method bias test

We gathered our data using a single respondent questionnaire, therefore there is a high possibility of common method bias (CMB) (see, Podsakoff et al. 2003; Ketokivi and Schroeder 2004). To counter this we have designed our questionnaire to reduce the effects of CMB, including using different scale formats and anchors for dependent and independent variables (see, Ketokivi and Schroeder 2004). We performed statistical analyses to assess the severity of CMB. Firstly, we conducted a traditional one-factor Harman’s test, as suggested by Podsakoff and Organ (1986), on the six constructs of our model. Results from this test showed that six constructs in the model are present and the most covariance explained by one factor is 18.63%, indicating that CMB is not a serious concern in our study. Secondly, we tested for CMB using the correlation marker technique guidelines advocated by Lindell and Whitney (2001). We found minimal differences between adjusted and unadjusted correlations. We also observed that the statistical significance of the correlations remained unaltered. Hence, based on these various statistical results, we argue that CMB is not present and a potential issue in our study.

### Hypothesis testing

Testing our research hypotheses using WarpPLS 7.0, which involves use of a bootstrapping procedure to estimate standard errors and the significance of the parameter estimates (Peng and Lai 2012), we report the values of PLS path co-efficient and the *p* values for the model (Table 4).

**Table 4:** Summary of hypotheses testing (N = 147)

Hypothesis	Beta coefficients	p	Supported/not supported
H1: IS→SCV	0.19	< 0.05	Supported
H2: IS→ ST	0.32	< 0.05	Supported
H3: SCV→ST	0.22	< 0.05	Supported
H4: ST→C	0.55	< 0.001	Supported
H5: ST→CO	0.67	< 0.001	Supported
H6: C→CO	0.46	< 0.001	Supported
H7: C→AG	0.71	< 0.001	Supported
H8: CO→AG	0.57	< 0.001	Supported

*IS* information sharing, *SCV* supply chain visibility, *ST* swift-trust, *C* commitment, *CO* collaboration, *AG* agility

H1 (IS → SCV) is supported (β = 0.19; *p* < 0.05), which is consistent with previous findings (see, Barratt and Oke 2007; Brandon-Jones et al. 2014). Similarly, H2 (IS→ST) is supported (β = 0.32; *p* < 0.05), suggesting that information sharing is positively associated with swift-trust and, again, this is consistent with previous findings (see, Dubey et al. 2019a). H3 (SCV → ST) is positively supported (β = 0.22; *p* < 0.05), suggesting that SCV is a positive predictor of swift-trust. H4 (ST → C) (β = 0.55; *p* < 0.001), H5 (ST → CO) (β = 0.67;  *p* < 0.001) and H6 (C → CO) (β = 0.46; *p* < 0.001) are all supported. These results support the premise that ST is a strong predictor of commitment and collaboration amongst the actors engaged in disaster relief efforts. Finally, H7 (C → AG) (β = 0.71; *p* < 0.001) and H8 (CO→AG) (β = 0.57; *p* < 0.001) are supported, suggesting that commitment and collaboration are strong predictors of agility in the humanitarian supply chain. These results paint a revealing picture of the various associations between swift-trust, commitment, collaboration and agility.

Next, we report the R², Q² and F² values, shown in Table 5. The R² value explains the explanatory power of the endogenous constructs. The R² for ST and AG are 0.32 and 0.67 respectively, which are moderately strong (Chin 1998). We also reported the effect size (F²) of each predictor. Based on the Cohen F² formula (Cohen 1988) the F² values of 0.35, 0.15 and 0.02 are considered large, medium and small respectively. Furthermore, we report the Q² values, which indicate the model’s capability to predict (Peng and Lai 2012). In our case we found that the model is a strong predictor of ST and AG.

**Table 5:** R², Prediction and Effect Size (N = 147)

Construct	R²	Q²	F² in relation to	
			ST	AG
IS	–		0.11	
SCV			0.09	
ST	0.32	0.21		
C				0.14
CO				0.17
AG	0.67	0.45		

## Discussion

Our investigation of the role of swift trust, commitment and collaboration in building agility, in order to move efficiently and effectively the disaster relief materials in humanitarian supply chains, focused upon three behavioral aspects of supply chain networks. Firstly, the role of information sharing in humanitarian operations. Altay and Pal (2014) argue that effective and efficient information flows facilitate a smooth response to disasters. However, the link between information sharing, supply chain visibility and swift-trust remains unexplored, see, Tatham and Kovács (2010) and Dubey et al. (2019a). Secondly, to improve collaboration among the humanitarian actors, the elements of swift-trust and commitment play a significant role. Here we sought to corroborate Trust-Commitment theory (Morgan and Hunt 1994) in the context of humanitarian settings. Thirdly, how swift-trust, commitment and collaboration help to explain agility in humanitarian supply chains is not well understood in humanitarian settings. The current humanitarian supply chain literature is at a nascent stage. Under these circumstances we argue that the integration of OIPT and RV provides a useful theoretical lens through which we can see how the individual elements of information sharing, supply chain visibility, swift-trust, commitment and collaboration interact with each other and combine to enhance agility in humanitarian supply chains. In doing so we draw a more complete picture of this topic than that presented in the previous literature. Bringing all the various interactions together into one holistic theoretical framework, we believe, enables some useful implications for practice, as well as raising some interesting research questions that may open new area for debates.

### Theoretical contributions

The role of networks in improving collaboration amongst actors is well discussed in the organizational literature (Cao and Zhang 2011). Likewise, the role of information diffusion for improving disaster response is well discussed in humanitarian literature (Altay and Pal 2014; Dubey et al. 2019a). What is less understood is how OIPT and RV together influence agility in humanitarian supply chains. In this respect two key aspects of our study signify its contribution to advancing a theory of agility in the context of humanitarian supply chain networks. Firstly, we have attempted to explain swift trust using the theoretical lens of OIPT. Hence we provide theory-driven empirical results, which further strengthens and corroborates previous work by demonstrating how information sharing and visibility, together, can explain swift-trust amongst the actors engaged in disaster relief efforts. Secondly, by utilizing RV, we add understanding of how to improve responses to humanitarian disasters, which is a missing link in the humanitarian supply chain literature. Although Moshtari (2016) attempted to explain the role of collaboration using RV, the relationships between swift-trust, commitment, collaboration and agility, which are analyzed in our study, have not been rigorously discussed so far in the literature.

Our research findings extend the work of Charles et al. (2010), who discussed how agility can be defined using the humanitarian experience. However, what is absent from the literature is insights which can offer explanations as to how to build agility into the humanitarian supply chain, which prior studies have shown to be a desirable characteristic. The current literature argues for the need to build agility in humanitarian supply chain networks, whilst at the same to explaining how to measure agility; see, for example, Charles et al. (2010) and Dubey and Gunasekaran (2016). Hence, in response to this, we claim that by integrating OIPT and RV we explain some unanswered questions. We further demonstrate how integration of these two independent organizational theories can provide a fresh perspective to explain some complex operational issues that are often ignored in real life situations.

### Managerial implications

Our findings offer practical guidance to those organizations involved in disaster relief activities. We provide an insight into building swift-trust in rapidly formed social networks involving actors in humanitarian supply chains. The role of swift-trust has been well recognized in the humanitarian literature. However, existing studies have largely focused on long-term relationships with less focus on the temporary relationships typical formed in humanitarian operations.

In recent years the frequency of humanitarian disasters has increased significantly and, in order to minimize the negative effects of such disasters on human lives, the need to form temporary relationships has increased significantly. In turn this has created a need for building swift-trust. However, due to the paucity of adequate studies, the disaster relief organizations have often struggled to address trust-related issues, which is an important reason for the failure of such disaster relief efforts. Our findings give practitioners a road map, whereby swift trust may influence agility, which is one of the most desirable characteristics to establishing effective and efficient humanitarian supply chains, via the creation of commitment and collaboration.

Previous works have studied how use of emerging technologies may influence agility. However, despite having strategic resources, due to a lack of swift-trust, commitment and collaboration among the humanitarian actors, disaster relief organizations have often failed to enhance their level of agility. Such a lack of agility hinders humanitarian organization’s responses to disasters and adversely impacts the supply chain’s ability to deliver appropriate relief materials to affected victims in a timely manner. For instance, COVID-19 has clearly exposed the failure of most advanced countries in the world to provide relief to all its victims i.e. care homes, the elderly, those in poverty. The failure of these advanced countries: poor speed, poor sensing-making capabilities and poor flexibility, is in part due to lack of trust, missing commitment and poor collaboration among government, health agencies, NGOs, necessary items manufacturing companies and logistics service providers. Our findings provide a better understanding of the subtle interplay of these relational values, which are often ignored during disaster relief efforts.

Finally, the findings of our study provides useful insights to those managers involved in building their commercial supply chain network to deal with uncertainties which threaten to disrupt and disable its functioning. In this respect the study enhances understanding of the information sharing mechanisms which may influence the lowering of behavioral uncertainty which is a critical challenge in real life supply chain management scenarios such as providing equipment, medicines and materials to deal with the COVID-19 pandemic.

## Conclusion, limitations and further research directions

Drawing broadly on OIPT, RV and the literature on swift-trust in humanitarian supply chain, we have developed a theoretical framework to explain agility. This framework was empirically tested using survey data. Our framework reconciles the independent contributions of two well established streams in management literature: (1) studies that focus on the impact of information sharing and supply chain visibility to explain swift trust and (2) studies that analyze how swift-trust and commitment can improve collaboration in social networks and, hence, explain how these relational values improve agility. We contribute to the humanitarian supply chain literature by focusing on a neglected area of swift trust and by extending and enriching the literature on humanitarian agility. Our research confirms the usefulness of OIPT and RV in explaining agility in humanitarian supply chain networks, highlighting the important role of swift trust.

Whilst we believe we have developed a sound and rich theoretical model, tested with a reliable instrument and data, our study has some limitations and unanswered questions remain. Our study was confined to respondents from the Indian sub-continent. Therefore, we are limited in term of any claims to generalize our findings beyond this empirical context. Therefore, we recommend data is collected using reliable databases from a wider range of international various organizations in order to compare findings with our results. Our work, being based upon OIPT and RV, did not take account of a learning perspective where each actor in the social network can share their expertise in order to adapt to a given situation. It is likely that over time, learning aspects will have an effect on supply agility in the humanitarian supply chain context. Hence we suggest future work is needed to extend our framework through the testing of additional constructs, such as experience in disaster-related projects or humanitarian operations skills. Finally, we have used a rationalistic approach in our study, though during an extensive review of the literature we realized that there is an urgent need for theory building in the context of humanitarian supply chains. In such situations the rationalist approach has its own limitations. Alternative research methods, such as case study, grounded theory, appreciative inquiry and action research may be useful to address the humanitarian supply chain problem from different perspectives and gain further insights that will help save live

## References

[CR1] Akter, S., D’Ambra, J., & Ray, P. (2011). An evaluation of PLS based complex models: The roles of power analysis, predictive relevance and GoF index. In *Proceedings of the 17th Americas conference on information systems (AMCIS2011)* (pp.&nbsp;1–7). Detroit: Association for Information Systems.

[CR2] Akter S, Wamba FS (2019). Big data and disaster management: A systematic review and agenda for future research. Annals of Operations Research.

[CR3] Akter S, Wamba F, Dewan S (2017). Why PLS-SEM is suitable for complex modelling? An empirical illustration in big data analytics quality. Production Planning & Control.

[CR4] Altay N, Gunasekaran A, Dubey R, Childe SJ (2018). Agility and resilience as antecedents of supply chain performance under moderating effects of organizational culture within the humanitarian setting: A dynamic capability view. Production Planning & Control.

[CR122] Altay N, Labonte M (2014). Challenges in humanitarian information management and exchange: Evidence from Haiti. Disasters.

[CR5] Altay N, Pal R (2014). Information diffusion among agents: Implications for humanitarian operations. Production and Operations Management.

[CR6] Armstrong JS, Overton TS (1977). Estimating nonresponse bias in mail surveys. Journal of Marketing Research.

[CR7] Balcik B, Beamon BM, Krejci CC, Muramatsu KM, Ramirez M (2010). Coordination in humanitarian relief chains: Practices, challenges and opportunities. International Journal of Production Economics.

[CR8] Banomyong R, Varadejsatitwong P, Oloruntoba R (2019). A systematic review of humanitarian operations, humanitarian logistics and humanitarian supply chain performance literature 2005 to 2016. Annals of Operations Research.

[CR9] Barratt M, Barratt R (2011). Exploring internal and external supply chain linkages: Evidence from the field. Journal of Operations Management.

[CR10] Barratt M, Oke A (2007). Antecedents of supply chain visibility in retail supply chains: A resource-based theory perspective. Journal of Operations Management.

[CR11] Behl A, Dutta P (2019). Humanitarian supply chain management: A thematic literature review and future directions of research. Annals of Operations Research.

[CR12] Blome C, Schoenherr T, Rexhausen D (2013). Antecedents and enablers of supply chain agility and its effect on performance: A dynamic capabilities perspective. International Journal of Production Research.

[CR13] Brandon-Jones E, Squire B, Autry CW, Petersen KJ (2014). A contingent resource‐based perspective of supply chain resilience and robustness. Journal of Supply Chain Management.

[CR14] Brusset X (2016). Does supply chain visibility enhance agility?. International Journal of Production Economics.

[CR15] Çankaya E, Ekici A, Özener O (2019). Humanitarian relief supplies distribution: An application of inventory routing problem. Annals of Operations Research.

[CR16] Cao M, Zhang Q (2011). Supply chain collaboration: Impact on collaborative advantage and firm performance. Journal of Operations Management.

[CR17] Caridi M, Crippa L, Perego A, Sianesi A, Tumino A (2010). Do virtuality and complexity affect supply chain visibility?. International Journal of Production Economics.

[CR18] Charles A, Lauras M, Van Wassenhove L (2010). A model to define and assess the agility of supply chains: Building on humanitarian experience. International Journal of Physical Distribution & Logistics Management.

[CR19] Chen DQ, Preston DS, Xia W (2013). Enhancing hospital supply chain performance: A relational view and empirical test. Journal of Operations Management.

[CR20] Chen IJ, Paulraj A (2004). Towards a theory of supply chain management: the constructs and measurements. Journal of Operations Management.

[CR21] Chen JV, Yen DC, Rajkumar TM, Tomochko NA (2011). The antecedent factors on trust and commitment in supply chain relationships. Computer Standards & Interfaces.

[CR22] Chin WW (1998). The partial least squares approach to structural equation modeling. Modern Methods for Business Research.

[CR23] Christopher M (2000). The agile supply chain: Competing in volatile markets. Industrial Marketing Management.

[CR25] Cohen J (1988). Statistical power analysis for the behavioral sciences.

[CR26] Cohen J (1992). Statistical power analysis. Current Directions in Psychological Science.

[CR27] Conway T, Swift JS (2000). International relationship marketing: The importance of psychic distance. European Journal of Marketing.

[CR28] Cozzolino A, Rossi S, Conforti A (2012). Agile and lean principles in the humanitarian supply chain: the case of the United Nations world food programme. Journal of Humanitarian Logistics and Supply Chain Management.

[CR29] Day JM, Melnyk SA, Larson PD, Davis EW, Whybark DC (2012). Humanitarian and disaster relief supply chains: A matter of life and death. Journal of Supply Chain Management.

[CR30] De Camargo JA, Mendonça PSM, de Oliveira JHC, Jabbour CJC, de Jabbour S (2019). Giving voice to the silent: A framework for understanding stakeholders’ participation in socially-oriented initiatives, community-based actions and humanitarian operations projects. Annals of Operations Research.

[CR31] Dubey R, Altay N, Blome C (2019). Swift trust and commitment: The missing links for humanitarian supply chain coordination?. Annals of Operations Research.

[CR32] Dubey R, Gunasekaran A (2016). The sustainable humanitarian supply chain design: agility, adaptability and alignment. International Journal of Logistics Research and Applications.

[CR33] Dubey R, Gunasekaran A, Bryde DJ, Dwivedi YK, Papadopoulos T (2020). Blockchain technology for enhancing swift-trust, collaboration and resilience within a humanitarian supply chain setting. International Journal of Production Research.

[CR34] Dubey R, Gunasekaran A, Childe SJ, Roubaud D, Wamba SF, Giannakis M, Foropon C (2019). Big data analytics and organizational culture as complements to swift trust and collaborative performance in the humanitarian supply chain. International Journal of Production Economics.

[CR35] Dubey R, Gunasekaran A, Papadopoulos T (2019). Disaster relief operations: Past, present and future. Annals of Operations Research.

[CR36] Dubey R, Luo Z, Gunasekaran A, Akter S, Hazen BT, Douglas MA (2018). Big data and predictive analytics in humanitarian supply chains. The International Journal of Logistics Management.

[CR37] Dwivedi YK, Shareef MA, Mukerji B, Rana NP, Kapoor KK (2018). Involvement in emergency supply chain for disaster management: A cognitive dissonance perspective. International Journal of Production Research.

[CR38] Dyer JH, Singh H (1998). The relational view: Cooperative strategy and sources of interorganizational competitive advantage. Academy of Management Review.

[CR39] Fawcett SE, Osterhaus P, Magnan GM, Brau JC, McCarter MW (2007). Information sharing and supply chain performance: The role of connectivity and willingness. Supply Chain Management: An International Journal.

[CR41] Foss Wamba S, Edwards A, Akter S (2019). Social media adoption and use for improved emergency services operations: The case of the NSW SES. Annals of Operations Research.

[CR42] Fosso Wamba S (2020). Humanitarian supply chain: A bibliometric analysis and future research directions. Annals of Operations Research.

[CR43] Galbraith JR (1973). Designing complex organizations.

[CR44] Gattiker TF, Goodhue DL (2004). Understanding the local-level costs and benefits of ERP through organizational information processing theory. Information & Management.

[CR45] Gunasekaran A, Dubey R, Fosso Wamba S, Papadopoulos T, Hazen BT, Ngai EW (2018). Bridging humanitarian operations management and organisational theory. International Journal of Production Research.

[CR46] Gunasekaran A, Papadopoulos T, Dubey R, Wamba SF, Childe SJ, Hazen B, Akter S (2017). Big data and predictive analytics for supply chain and organizational performance. Journal of Business Research.

[CR47] Gupta S, Altay N, Luo Z (2019). Big data in humanitarian supply chain management: A review and further research directions. Annals of Operations Research.

[CR49] Herlin H, Pazirandeh A (2012). Nonprofit organizations shaping the market of supplies. International Journal of Production Economics.

[CR50] Herlin H, Pazirandeh A (2015). Avoiding the pitfalls of cooperative purchasing through control and coordination: Insights from a humanitarian context. International Journal of Procurement Management.

[CR51] Hitt MA, Keats BW, DeMarie SM (1998). Navigating in the new competitive landscape: Building strategic flexibility and competitive advantage in the 21st century. Academy of Management Perspectives.

[CR52] Hocutt MA (1998). Relationship dissolution model: Antecedents of relationship commitment and the likelihood of dissolving a relationship. International Journal of Service Industry Management.

[CR53] Holguín-Veras J, Jaller M, Van Wassenhove LN, Pérez N, Wachtendorf T (2012). On the unique features of post-disaster humanitarian logistics. Journal of Operations Management.

[CR54] Hsu CC, Kannan VR, Tan KC, Leong K (2008). Information sharing, buyer–supplier relationships, and firm performance: A multi-region analysis. International Journal of Physical Distribution & Logistics Management.

[CR55] Hung, Y.-T. C., Dennis, A. R., & Robert, L. (2004). Trust in virtual teams: Towards an integrative model of trust formation. In *Proceedings of the 37th Hawaii international conference on systems sciences, track 1* (Vol.&nbsp;1).

[CR56] Ivanov D (2020). Viable supply chain model: Integrating agility, resilience and sustainability perspectives—Lessons from and thinking beyond the COVID-19 pandemic. Annals of Operations Research.

[CR57] Ivanov D, Dolgui A (2020). Viability of intertwined supply networks: Extending the supply chain resilience angles towards survivability. A position paper motivated by COVID-19 outbreak. International Journal of Production Research.

[CR58] Jabbour CJC, Sobreiro VA, de Sousa Jabbour ABL, de Souza Campos LM, Mariano EB, Renwick DWS (2019). An analysis of the literature on humanitarian logistics and supply chain management: Paving the way for future studies. Annals of Operations Research.

[CR126] Jin Y, Fawcett AM, Fawcett SE (2013). Awarenes is not enough. International Journal of Physical Distribution & Logistics Management.

[CR59] Kabra G, Ramesh A (2016). Information technology, mutual trust, flexibility, agility, adaptability: Understanding their linkages and impact on humanitarian supply chain management performance. Risk, Hazards & Crisis in Public Policy.

[CR60] Ketokivi MA, Schroeder RG (2004). Perceptual measures of performance: Fact or fiction?. Journal of Operations Management.

[CR62] Kock N (2019). From composites to factors: Bridging the gap between PLS and covariance-based structural equation modelling. Information Systems Journal.

[CR64] Kwon IWG, Suh T (2004). Factors affecting the level of trust and commitment in supply chain relationships. Journal of Supply Chain Management.

[CR125] Kwon IWG, Suh T (2005). Trust, commitment and relationships in supply chain management: A path analysis. Supply chain management: An international journal.

[CR65] Lee HL (2004). The triple-A supply chain. Harvard business review.

[CR66] Lee HL, Padmanabhan V, Whang S (1997). Information distortion in a supply chain: The bullwhip effect. Management Science.

[CR67] L’Hermitte C, Tatham P, Brooks B, Bowles M (2016). Supply chain agility in humanitarian protracted operations. Journal of Humanitarian Logistics and Supply Chain Management.

[CR68] Li S, Lin B (2006). Accessing information sharing and information quality in supply chain management. Decision Support Systems.

[CR70] Lindell MK, Whitney DJ (2001). Accounting for common method variance in cross-sectional research designs. Journal of Applied Psychology.

[CR71] Lu Y, Ramamurthy K (2011). Understanding the link between information technology capability and organizational agility: An empirical examination. MIS Quarterly.

[CR72] Maghsoudi A, Pazirandeh A (2016). Visibility, resource sharing and performance in supply chain relationships: Insights from humanitarian practitioners. Supply Chain Management: An International Journal.

[CR73] McLaren M, Loosemore M (2019). Swift trust formation in multi-national disaster project management teams. International Journal of Project Management.

[CR74] Meyerson D, Weick KE, Kramer RM, Kramer RM, Tyler TR (1996). Swift trust and temporary groups. Trust in organizations: Frontiers of theory and research.

[CR76] Modgil S, Singh RK, Foropon C (2020). Quality management in humanitarian operations and disaster relief management: A review and future research directions. Annals of Operations Research.

[CR77] Morgan RM, Hunt SD (1994). The commitment-trust theory of relationship marketing. The Journal of Marketing.

[CR78] Moshtari M (2016). Inter-organizational fit, relationship management capability, and collaborative performance within a humanitarian setting. Production and Operations Management.

[CR79] Motamarri S, Akter S, Yanamandram V (2020). Frontline employee empowerment: Scale development and validation using Confirmatory Composite Analysis. International Journal of Information Management.

[CR80] Narayanan S, Narasimhan R, Schoenherr T (2015). Assessing the contingent effects of collaboration on agility performance in buyer–supplier relationships. Journal of Operations Management.

[CR81] Oloruntoba R, Gray R (2006). Humanitarian aid: An agile supply chain?. Supply Chain Management: An International Journal.

[CR82] Oloruntoba R, Hossain GF, Wagner B (2019). Theory in humanitarian operations research. Annals of Operations Research.

[CR83] Oloruntoba R, Kovács G (2015). A commentary on agility in humanitarian aid supply chains. Supply Chain Management: An International Journal.

[CR84] Papadopoulos T, Gunasekaran A, Dubey R, Altay N, Childe SJ, Fosso-Wamba S (2017). The role of big data in explaining disaster resilience in supply chains for sustainability. Journal of Cleaner Production.

[CR123] Peng DX, Lai F (2012). Using partial least squares in operations management research: A practical guideline and summary of past research. Journal of Operations Management.

[CR85] Podsakoff PM, MacKenzie SB, Lee JY, Podsakoff NP (2003). Common method biases in behavioral research: A critical review of the literature and recommended remedies. Journal of Applied Psychology.

[CR86] Podsakoff PM, Organ DW (1986). Self-reports in organizational research: Problems and prospects. Journal of Management.

[CR87] Prajogo D, Olhager J (2012). Supply chain integration and performance: The effects of long-term relationships, information technology and sharing, and logistics integration. International Journal of Production Economics.

[CR88] Prasad S, Zakaria R, Altay N (2018). Big data in humanitarian supply chain networks: A resource dependence perspective. Annals of Operations Research.

[CR89] Prasanna SR, Haavisto I (2018). Collaboration in humanitarian supply chains: An organisational culture framework. International Journal of Production Research.

[CR90] Premkumar GKWR, King WR (1994). Organizational characteristics and information systems planning: An empirical study. Information Systems Research.

[CR91] Queiroz MM, Ivanov D, Dolgui A, Wamba SF (2020). Impacts of epidemic outbreaks on supply chains: mapping a research agenda amid the COVID-19 pandemic through a structured literature review. Annals of Operations Research.

[CR92] Sambamurthy V, Bharadwaj A, Grover V (2003). Shaping agility through digital options: Reconceptualizing the role of information technology in contemporary firms. MIS quarterly.

[CR93] Schiffling S, Hannibal C, Fan Y, Tickle M (2020). Coopetition in temporary contexts: Examining swift trust and swift distrust in humanitarian operations. International Journal of Operations & Production Management.

[CR94] Schiffling S, Hannibal C, Tickle M, Fan Y (2020). The implications of complexity for humanitarian logistics: A complex adaptive systems perspective. Annals of Operations Research.

[CR95] Seybolt TB (2009). Harmonizing the humanitarian aid network: Adaptive change in a complex system. International Studies Quarterly.

[CR96] Sezen B (2008). Relative effects of design, integration and information sharing on supply chain performance. Supply Chain Management: An International Journal.

[CR97] Shareef MA, Dwivedi YK, Mahmud R, Wright A, Rahman MM, Kizgin H, Rana NP (2019). Disaster management in Bangladesh: developing an effective emergency supply chain network. Annals of Operations Research.

[CR98] Sherehiy B, Karwowski W, Layer JK (2007). A review of enterprise agility: Concepts, frameworks, and attributes. International Journal of Industrial Ergonomics.

[CR99] Simatupang TM, Sridharan R (2002). The collaborative supply chain. The International Journal of Logistics Management.

[CR100] Simatupang TM, Sridharan R (2011). A drama theory analysis of supply chain collaboration. International Journal of Collaborative Enterprise.

[CR101] Smith KG, Grimm CM, Gannon MJ, Chen MJ (1991). Organizational information processing, competitive responses, and performance in the US domestic airline industry. Academy of Management journal.

[CR102] Sodhi MS, Tang CS (2019). Research opportunities in supply chain transparency. Production and Operations Management.

[CR103] Srinivasan R, Swink M (2015). Leveraging supply chain integration through planning comprehensiveness: An organizational information processing theory perspective. Decision Sciences.

[CR104] Srinivasan R, Swink M (2018). An investigation of visibility and flexibility as complements to supply chain analytics: An organizational information processing theory perspective. Production and Operations Management.

[CR105] Stewart M, Ivanov D (2019). Design redundancy in agile and resilient humanitarian supply chains. Annals of Operations Research.

[CR106] Swanson RD, Smith RJ (2013). A path to a public–private partnership: Commercial logistics concepts applied to disaster response. Journal of Business Logistics.

[CR107] Tang CS (2006). Perspectives in supply chain risk management. International Journal of Production Economics.

[CR108] Tatham P, Kovács G (2010). The application of “swift trust” to humanitarian logistics. International Journal of Production Economics.

[CR124] Vijayasarathy LR (2010). Supply integration: An investigation of its multi-dimensionality and relational antecedents. International Journal of Production Economics.

[CR109] Wagner SM, Thakur-Weigold B (2018). Supporting collaboration in humanitarian supply chains–insights from a design science project. Production Planning & Control.

[CR110] Wagner SM, Thakur-Weigold B, Gatti F, Stumpf J (2020). Measuring and improving the impact of humanitarian logistics consulting. Production Planning & Control.

[CR111] Wang ET, Wei HL (2007). Interorganizational governance value creation: Coordinating for information visibility and flexibility in supply chains. Decision Sciences.

[CR113] Wieland A, Wallenburg CM (2013). The influence of relational competencies on supply chain resilience: A relational view. International Journal of Physical Distribution & Logistics Management.

[CR114] Wilson DT (1995). An integrated model of buyer–seller relationships. Journal of the Academy of Marketing Science.

[CR116] Wu WY, Chiang CY, Wu YJ, Tu HJ (2004). The influencing factors of commitment and business integration on supply chain management. Industrial Management & Data Systems.

[CR117] Yang J (2014). Supply chain agility: Securing performance for Chinese manufacturers. International Journal of Production Economics.

[CR118] Yigitbasioglu OM (2010). Information sharing with key suppliers: A transaction cost theory perspective. International Journal of Physical Distribution & Logistics Management.

[CR119] Yu Z, Yan H, Edwin Cheng TC (2001). Benefits of information sharing with supply chain partnerships. Industrial Management & Data Systems.

[CR120] Yusuf YY, Gunasekaran A, Musa A, Dauda M, El-Berishy NM, Cang S (2014). A relational study of supply chain agility, competitiveness and business performance in the oil and gas industry. International Journal of Production Economics.

[CR121] Zhou H, Benton WC (2007). Supply chain practice and information sharing. Journal of Operations management.

